# Editorial: Future directions and current trends in cellular therapies

**DOI:** 10.3389/fmed.2025.1732925

**Published:** 2025-11-12

**Authors:** Ruxandra Irimia, Eleni Gavriilaki

**Affiliations:** 1 Emergency Hospital Prof. Dr. Agrippa Ionescu, Bucharest, Romania; 2 Aristoteleio Panepistemio Thessalonikes, Thessaloniki, Greece

**Keywords:** autologous stem cell transplantation, allogeneic stem cell transplantation, CAR-T cell therapy, engineered immunity, stem cell transplant

Sparked by observations of marrow failure after massive irradiation during World War II, the earliest bone marrow infusions were attempted before the very concept of immunologic matching even existed. Almost simultaneously on both sides of the Atlantic, the pioneering efforts of Georges Mathé and E. Donnall Thomas explored whether transplanted marrow could restore hematopoiesis after lethal radiation exposure and, soon thereafter, serve as a treatment for acute leukemias.

These early attempts, followed by extensive animal experiments, demonstrated that durable engraftment required close histocompatibility between donor and recipient, thereby establishing the conceptual foundation for transplant matching. The discovery of the major histocompatibility complex by Jean Dausset, followed by the first experimental applications of HLA matching by Fritz Bach, enabled the first successful allogeneic procedure in a non-identical sibling. This breakthrough paved the way for the first unrelated donor transplant in 1973 and, a few years later in 1979, the first unrelated allogeneic bone marrow transplant performed for acute leukemia. As transplant success became more frequent, so too did recognition of its infectious and immune-mediated complications which in turn drove advances in HLA typing, graft manipulation, conditioning personalization, and prophylaxis against both infection and GVHD.

What followed over the next decades was not merely technical refinement, but a gradual reframing of the therapy's fundamental purpose, as an immunologic treatment rather than a simple hematopoietic rescue. Long before “cellular therapy” entered the lexicon, the experience showed that most durable remissions after transplant could not be explained by cytotoxic intensification but rather by the immunologic interaction between donor and host. The donor lymphocyte infusion was the clearest conceptual breakthrough: disease relapse could be re-treated not with chemotherapy, but by intensifying the immune component alone.DLI was therefore more than a salvage strategy; it was the first prototype of adoptive cellular therapy, and engineered immunity through CAR-T cells represented the logical maturation of the this therapeutic principle. CAR-T therapy is therefore not a conceptual departure from transplant, but as a refinement of its central mechanism.

Today, engineered T-cell therapies are widely used for the treatment of relapsed or refractory B-cell malignancies, including diffuse large B-cell lymphoma, mantle cell lymphoma, follicular lymphoma, as well as B-cell acute lymphoblastic leukemia (ALL). In myeloma, CAR-T therapies are moving earlier in the disease course, and current research is investigating whether autologous stem cell transplant still has a place in the modern management of this disease. This evolution prompts a practical question: will CAR-T therapies replace HSCT?

While CAR-T signals the next phase of cellular therapies, the daily clinical reality of transplantation reminds us that the field is still actively evolving. The current Research Topic crystallizes decades of accumulated experience in four real-world studies addressing core challenges of stem cell transplantation: infectious complications, post-transplant neoplasms, and post-relapse management.

The first paper published in the current Research Topic addresses letermovir prophylaxis for CMV in haploidentical HSCT recipients. Recent advances have shifted the preferred approach for cytomegalovirus prevention in allogeneic hematopoietic stem cell transplant recipients toward universal antiviral prophylaxis with letermovir for CMV-seropositive patients. The work of Huang et al. shows that although effective in preventing CMV viremia (32.1% vs. 46.2%), LTV prophylaxis was associated with increased EBV reactivation (38.7% vs.13.7%) and a higher rate of post-transplant disease relapse (13.2% vs. 6.1%). This appears to coincide with delayed and functionally compromised T-lymphocyte recovery, suggesting a potential immune-mediated mechanism of disease relapse.

The second study from Sun et al., expands the understanding of post-transplant viral enteritis by characterizing the most common pathogens involved. Beyond the well-recognized role of CMV, 13 additional pathogens were identified, with HHV-6 emerging as a far more common cause of viral enteritis than previously appreciated (37.3% of cases). Most cases occurred within 2–3 months post-HSCT, and co-infection was frequent, with over one quarter of patients harboring two or more viruses. CMV remained a major contributor (37.3% of cases) and, consistent with prior literature, was associated with inferior outcomes and increased non-relapse mortality.

The third work by Rihani et al. covers a well-recognized complication of transplantation, the risk of secondary neoplasms (SN). In their retrospective cohort of adult and pediatric patients, covering the period between January 2003 and December 2023, 1.6% of the patients developed a secondary malignancy. The majority of SNs were solid tumors (65.2%), with hematologic malignancies comprising 32.5%. The risk factors identified by the authors align with the existing literature and include the exposure to alkylating agents or topoisomerase II inhibitors, chronic GVHD and CMV reactivation.

Finally, the fourth work included in our Research Topic addresses one of the most challenging clinical scenarios: the management of relapse after allo-HSCT for high-risk myeloid malignancies. Cheng et al., describe the outcomes of a modern cohort of 106 AML patients relapsing after transplantation and demonstrate that the combination of venetoclax–hypomethylating agent strategy offers significantly superior outcomes compared with intensive chemotherapy. In their study, patients treated with the doublet combination achieved a significantly higher complete remission rate (56.6% vs. 26.4%), higher MRD negativity rates (70.0% vs. 35.7%) and extended median overall survival to 12.6 months vs. 5.8 months.

In conclusion, the landscape of cellular therapy is rapidly evolving, with engineered immune cell products increasingly shaping the future of curative treatment. The trajectory of innovation suggests a gradual narrowing of indications for classical stem cell transplantation as precision-engineered approaches expand. Yet, at present, HSCT remains an indispensable, lifesaving therapy. Ongoing progress in transplantation comes from deeper understanding and better prevention of short- and long-term complications, optimizing conditioning regimens, and improved supportive therapies. The future of the field will therefore not be defined by replacement, but by convergence: the continued evolution of transplantation and cellular engineering as complementary expressions of immune-based cure.

